# Clinical effects and adverse effects of intravenous lipid emulsion treatment in dogs and cats with suspected poisoning

**DOI:** 10.1371/journal.pone.0298828

**Published:** 2024-05-29

**Authors:** Dschaniena Kiwitz, Carina Markert, René Dörfelt

**Affiliations:** 1 Veterinary Clinic for Small Animals, Tierklinik Hofheim, Tierärzte IVC Evidensia GmbH, Hofheim am Taunus, Hessen, Germany; 2 LMU Small Animal Clinic, LMU München, München, Germany; Universidade Federal de Minas Gerais, BRAZIL

## Abstract

This retrospective study aimed to evaluate the effects on the clinical signs of poisoning and adverse effects of intravenous lipid emulsion treatment in 82 animals (dogs and cats) with suspected poisonings over 18 months. Physical examination parameters and state of consciousness were documented every hour after the intravenous administration of a bolus of 2 ml/kg and 0.25 ml/kg/min over 60 minutes of a 20% intravenous lipid emulsion. The modified Glasgow coma scale and laboratory findings (blood gas analysis, triglyceride, lactate) were evaluated initially and three hours after discontinuing intravenous lipid emulsion administration. A statistical evaluation of the occurrence of adverse effects and the development of laboratory values was performed. A decrease in respiratory rate in the second control (8–12 hours) after ILE was observed. Three hours after completing of the intravenous lipid emulsion, triglyceride concentration increased about 10 times (p <0.001). Venous carbon dioxide partial pressure, bicarbonate, base excess, as well as the electrolytes sodium, potassium and ionized calcium decreased significantly (p <0.001). Patients who experienced a worsening of the modified Glasgow coma scale had a higher increase in triglyceride concentrations (p = 0.041) and plasma lactate (p = 0.034) and a larger decrease in bicarbonate concentrations (p = 0.053) compared to others. About 54% (n = 44) of the patients showed adverse effects which could be attributed to the administration of intravenous lipid emulsion and may be associated with a higher triglyceride increase. All of them were completely reversible within 33 hours. Adverse effects associated with intravenous lipid emulsion therapy were observed in half of the patients and were associated with a higher increase in triglycerides.

## Introduction

Dogs and cats presenting with poisoning pose a challenge to the veterinarian. For most toxicants, an antidote or blood purification methods for toxin elimination are not available. Therefore, symptomatic treatment is indicated for most poisoned animals. In recent years, the administration of intravenous lipid emulsions (ILE) for the elimination of lipophilic toxicants has been increasingly used in veterinary medicine [1–3].

ILE is a mostly 20% oil-in-water emulsion, which, depending on the preparation, consists of various combinations of soybean, olive or sometimes fish oil as well as medium-length chain triglycerides, which is licensed for total parenteral nutrition in humans. The first case report of a dog with moxidectin intoxication successfully treated with ILE using a bolus of 2 ml/kg followed by a continuous rate infusion (CRI) of 3.75 ml/kg/h for 4 hours was published in 2009. The puppy was discharged after two days of hospitalization, without clinical abnormalities [4]. Since then, ILE has been used in multiple case reports and for different poisonings with different dosages in both dogs and cats [1, 3, 5, 6].

The mechanism of action of intravenous lipids for the treatment of poisonings is not yet fully understood. The most commonly described theory, based on several studies, is the "lipid shuttle" theory, but an improvement in myocardial performance is also conceivable [7–13]. The "lipid shuttle" describes the formation of an additional lipid compartment within the vascular space, into which lipophilic substances are bound, remain, and are transported to the liver, where toxicants are eliminated from the body. According to this theory, the target toxicant should have a high lipophilicity, which is described as the octanol-water partition coefficient of a substance and usually named log P. Substances with a log P >1.0 accumulate in the lipophilic emulsion, which may help to hasten elimination [9, 14, 15].

One prospective clinical study on cats with permethrin poisoning compared the effect of ILE administration at a dose of 0.25 ml/kg/h for 30–60 minutes with the same volume of isotonic saline after decontamination. Cats receiving ILE showed a faster improvement of clinical neurological signs like facial twitching, generalized tremor and seizures without relevant adverse effects compared to the control group [16]. In another prospective experimental study in rabbits with ivermectin poisoning an ILE Bolus of 2 ml/kg and a constant rate infusion of 0.25 ml/kg/min over 60 minutes was performed. The use of ILE lead to clinical improvement, and lead to hypertriglyceridemia, while biochemically and histologically safe [17].

Only a few veterinary publications are dealing with the adverse effects of ILE. Previously reported adverse effects include pruritus and corneal lipidosis in cats, red spots on the skin in pigs, swelling and pain in the area of the venous catheter and a decrease in hematocrit and hemoglobin concentration in dogs and hyperlipidemia in dogs and cats [1, 3, 5, 8, 16, 18, 19]. One dog was reported to have developed acute respiratory distress syndrome (ARDS) and died from that complication [20]. In addition, a fat overload syndrome has been described in human medicine, which manifests clinically with vomitus, tachypnea, dyspnea, pale mucous membranes, tachycardia, pyrexia, muscle pain, ecchymosis, hepato-splenomegaly and impaired judgment. Laboratory findings may include hyperlipidemia, lymphopenia, neutropenia, thrombocytopenia and hemostasis disorder [21–25]. The development of pancreatitis has also been described in humans [26].

Neurologic deficits such as lethargy, decreased level of consciousness, convulsions and coma have been described in case reports from human medicine after ILE treatment [22, 27, 28]. In veterinary medicine, one case series in cats with ibuprofen intoxication reports a cat that was stuporous for 16 hours immediately after administration of ILE. The cat recovered uneventfully after this period [29]. One retrospective analysis of ILE in 313 dogs and 100 cats examined the effects and adverse effects of ILE using multiple dosage schemes. Nevertheless, this study was unable to establish associations of ILE adverse events with laboratory abnormalities due to a lack of data [30].

In a small cohort of six dogs and two cats receiving ILE with an initial bolus of 2 ml/kg followed by a CRI of 0.25 ml/kg/h over 60 minutes one hour after ILE administration, an increase of triglycerides has been observed. Three hours after discontinuing ILE administration, serum triglycerides were measured again and were close to normal in 75% of the patients [31]. This suggests a potential connection between the complications observed in various case reports, including the occasional decreased mentation and the temporary increase in triglycerides. The aim of this retrospective study was to evaluate the effects on the clinical signs of poisoning and adverse effects of ILE treatment in dogs and cats with confirmed or suspected poisonings.

## Materials and methods

After the introduction of a standard treatment protocol for poisoning with ILE, the medical records of dogs and cats treated over 18 months at a veterinary referral hospital were retrospectively reviewed. Dogs and cats were included if they received ILE due to confirmed or suspected poisoning according to the in-house standard protocol for ILE administration as mentioned below. Treatment with ILE was initiated in patients poisoned with a confirmed or suspected lipophilic substance with a log P >1.0 or if they had severe clinical signs of poisoning, e.g., seizures. Patients were excluded if they were treated as an outpatient (i.e. not hospitalized), or if clinical signs were associated with a primary intracranial disease.

### Clinical examination and laboratory analysis

Data from physical examination findings (heart rate, respiratory rate, temperature, level of consciousness) and the modified Glasgow coma scale (MGCS) at presentation and during hospitalization were collected. Data from laboratory findings, including complete blood count (ProCyte Dx Hematology Analyzer, Idexx GmbH, Kornwestheim, Germany), and clinical chemistry with special attention to triglyceride (Cobas Integra 400 plus, Roche Diagnostic (Switzerland) AG, Rotkreuz ZG, Switzerland), lactate levels (Catalyst Dx Chemistry Analyzer, Idexx GmbH, Kornwestheim, Germany), and venous blood gas analysis (VetScan i-Stat1, Abraxis, Griesheim, Germany) were analyzed at time of admission and during hospitalization.

### Protocol

The in-house ILE protocol included ILE administration after decontamination via induced emesis (0.08 mg/kg apomorphine intramuscular for dogs, 0.15 μg/kg dexmedetomidine intramuscular for cats) in patients with a normal level of consciousness and if no contraindications for emesis are present. If there were contraindications of induced emesis and the ingestion of the toxicant was less than four hours ago a gastric lavage was performed. ILE is started immediately in patients with induced emesis and in those who received gastric lavage if symptoms persisted and consisted of a 20% ILE (Lipofundin MCT/LCT 20% from B. Braun, Melsungen) administered with 1.5 ml/kg in 2 minutes followed by 0.25 ml/kg over 60 minutes.

Clinical parameters, including heart rate, respiratory rate, temperature and level of consciousness were performed before ILE administration and every hour until three hours after cessation of ILE and the next day. In addition, the MGCS was evaluated initially and three hours after discontinuing ILE. Analysis of the venous blood gases, complete blood count, clinical chemistry, triglyceride, and lactate concentration was performed initially and three hours post-ILE treatment. If symptoms did not improve and triglyceride concentrations were not increased more than twofold, an additional bolus of ILE was indicated. Additional bolus was also administered if the animal showed severe clinical signs of the poisoning again.

### Statistical analysis

Data were recorded using a commercial software program (Excel, Microsoft Office 2016, Microsoft Corporation, Redmond, WA, USA) and were statistically analyzed (Prism 5, GraphPad, San Diego, CA, USA). Data were evaluated for normal distribution using the D’Agostino & Pearson normality test and are reported as median and range. The physical examination findings and laboratory parameters before and after ILE were compared using the Wilcoxon-matched pairs signed-rank test. The development of physical examination findings and laboratory parameters in relation to the course of the MGCS were evaluated using the MGCS the Kruskal-Wallis test with a Dunn’s multiple comparison test was used. To analyze a correlation between the decrease of MGCS in the development of physical examination and laboratory parameters a Chi-square test was used. To compare the x-fold triglyceride increase with the adverse effects the Kruskal-Wallis test with a Dunn’s multiple comparison test was applied.

## Results

Over the 18-month period, 82 animals (65 dogs and 17 cats) met the inclusion criteria. The dogs were median two years old (0.2–13.0 years) and had a mean body weight of 21.7 kg (1.3–54.0). The cats were median three years (0.3–14.0 years) old and had a mean body weight of 4.0 (1.8–6.2) kg. The median time from intoxication to the first clinical signs was 3.0 hours (0.5–7.0 hours) in dogs and 6.0 hours (1.0–8.0 hours) in cats. The median time from ingestion of the toxicant to presentation was 6 hours (1–10 hours) in dogs and 8 hours (1–24 hours) in cats. Median hospitalization time was 17 hours (5–56 hours) for the dogs. All dogs survived to discharge. Median hospitalization time for cats was 25 hours (6–63 hours), with one non-surviving cat (Table 1).

**Table 1:** General data, time from toxicant uptake to clinical signs and presentation, hospitalization time and outcome of 65 dogs and 17 cats with confirmed and suspected poisonings treated with intravenous lipid therapy.

Parameter	dogs	cats
Age (years)	2 (0.2–13.0)	3.0 (0.3–14.0)
Weight (kg)	21.7 (1.3–54.0)	4.0 (1.8–6.2)
Duration of toxicant uptake until clinical signs (hours)	3.0 (0.5–7.0)	6.0 (1.0–8.0)
Duration from intoxication to presentation (hours)	6 (1–10)	8 (1–24)
Duration from onset of clinical sings to presentation (hours)	2.0 (0.5–8.0)	2.0 (1.0–16.0)
Hospitalisation time (hours)	17 (5–56)	25 (6–63)
Outcome (discharge/death)	65/0	16/1

The toxicant was not identified in 51 patients (62%). Ingestion of alpha-chloralose and tremorgenic mycotoxins for every seven patients, were observed most frequently in the canine population. Three dogs ingested tetrahydrocannabinol (THC) and one each ingested *Taxus baccata*, carprofen, metaldehyde, permethrin, ivermectin, and 3,4-methylenedioxy-N-methylamphetamine (MDMA). The toxicant ingested in nine cats was not identified. Alpha-chloralose was ingested in four cats and THC, *Taxus baccata*, carprofen and ibuprofen was ingested in 1 cat each (Table 2).

**Table 2:** Suspected or confirmed toxicants in 65 dogs and 17 cats treated with intravenous lipid therapy with clinical signs or a history of poisoning.

Poison	Dogs (n)	Cats (n)
Alpha-chloralose	7/65	4/17
Carprofen	1/65	1/17
Ibuprofen		1/17
Ivermectin	1/65	
3,4-methylenedioxy-N-methylamphetamine	1/65	
Metaldehyd	1/65	
Permethrin	1/65	
*Taxus baccata*	1/65	1/17
Tetrahydrocannabinol	3/65	1/17
Tremorogenic mycotoxins	7/65	
Unknown	42/65	9/17

n: number of animals affected.

Clinical parameters, state of consciousness and MGCS assessed retrospectively at time of initiation (time 0) and three hours after ILE. After the administration of ILE heart rate in both dogs and cats and temperature in cats remained stable while the respiratory rate decreased (Table 3). A significant difference in temperature was observed in dogs to the baseline. The level of consciousness and MGCS did not change (Table 4).

**Table 3:** Clinical examination parameters before and after starting the administration of intravenous lipid emulsion treatment for poisoning in dogs and cats with clinical signs or a history of poisoning.

	Parameter	n	Before ILE	3 hours after ILE	8–12 hours after ILE	p
Dogs	Heart rate (bpm)	62	100 (45–180)	100 (60–176)	100 (64–160)	0.511
	Respiratory rate (breaths/min)	62	16 (16–120)	24 (12–156)*	16 (16–120) ^#^	<0.001
	Temperature (°C)	61	38.6 (35.6–40.4)	38.5 (35.7–39.8)	38.3 (36.6–39.0)*	0.002
Cats	Heart rate (bpm)	16	179 (120–200)	159 (106–200)	180 (120–180)	0.208
	Respiratory rate (breaths/min)	16	16 (16–52)	24 (16–60)*	16 (16–48) ^#^	0.007
	Temperature (°C)	16	38.4 (36.1–39.1)	38.0 (36.7–38.7)	38.5 (37.2–40.9)	0.165

bpm: beats per minute; p: significance level.

* significant different baseline

^#^ significant different to 3 hours after ILE

n: number of animals

**Table 4:** Consciousness and modified Glasgow coma scale before and after starting the administration of intravenous lipid emulsion treatment in 65 dogs and 17 cats with clinical signs or a history of poisoning.

Parameter	n	Before ILE	3 hours after ILE	p
Level of consciousness*	70	2 (1–4)	2 (1–4)	0.514
MGCS	82	16 (7–18)	17 (5–18)	0.307

n: number of animals

* Level of consciousness: 1 = undisturbed, 2 = apathy, 3 = stupor, 4 = coma

MGCS: modified Glasgow coma scale; p: significance level.

Three hours post-ILE, triglyceride concentration increased about 10 times (p <0.001). Venous carbon dioxide partial pressure (pCO_2_), bicarbonate, base excess, as well as the electrolytes sodium, potassium and ionized calcium decreased significantly (p <0.001 for all; Table 5).

**Table 5:** Laboratory parameters (triglycerides, lactate, venous blood gas analysis) of 65 dogs and 17 cats at the time of administration and 3 hours after the end of intravenous lipid emulsion (ILE) for treatment of intoxications.

Parameter (unit)	n	Before ILE	3 h after ILE	p
Trig (mmol/l)	80	0.58 (0.11–10.00)	5.65 (0.31–117.40)	< 0.001*
pH	75	7.35 (7.11–7.64)	7.35 (7.20–7.70)	0.874
pCO_2_ (mmHg)	74	42.4 (23.6–74.5)	35.7 (21.1–59.2)	< 0.001*
HCO_3_^-^ (mmol/l)	74	23.3 (15.7–34.4)	20.5 (11.2–28.0)	< 0.001*
BE (mmol/l)	75	-3.0 (-9.0–7.0)	5.0 (-15.0–2.0)	< 0.001*
Na (mmol/l)	74	146 (141–156)	144 (134–154)	< 0.001*
K (mmol/l)	75	4.0 (1.4–5.3)	3.6 (2,6–5,1)	< 0.001*
iCa (mmol/l)	74	1.39 (1.15–1.51)	1.33 (1.06–1.56)	< 0.001*
Lac (mmol/l)	75	1.7 (0.5–7.6)	2.0 (0.5–10.6)	0.372
Hb (mmol/l)	72	14.5 (6.1–22.3)	15.0 (8.2–19.7)	0.246
HCT (%)	72	42.5 (18.0–71.0)	44.0 (24.0–58.0)	0.434

n: number of all 65 dogs and 17 cats from which the parameter was available in the medical records. p: significance level; Trig: triglycerides; HCO_3_^-^: bicarbonate; BE: base excess; Na: sodium; K: potassium; iCa: ionised calcium; Lac: lactate; Hb: Hemoglobin; HCT: hematocrit; pCO_2_: carbon dioxide partial pressure

* significant changes

No significant difference in changes in physical examination findings before and after ILE treatment was observed between patients with improving, stable or worsening MGCS. However, patients who experienced a worsening of the MGCS after ILE treatment had a higher increase in triglyceride concentrations (p = 0.041) and plasma lactate (p = 0.034) concentrations compared to others. A stronger decrease in ionized calcium was observed in patients with both improvement and deterioration of the MGCS compared to patients with a constant MGCS (p = 0.002; Table 6).

**Table 6:** Changes of physical examination and laboratory findings (triglycerides, lactate, venous blood gas analysis) as median (minimum–maximum) between the administration and 3 hours after the end of intravenous lipid emulsion (ILE) for treatment of poisonings in 65 dogs and 17 cats in relation to the course of the modified Glasgow coma scale (MGCS).

Parameter	MGCS improved		MGCS equal		MGCS worsened		p
	Value	n	value	n	value	n	
**Heart rate (bpm)**	-1,5 (-50–64)	34	-2 (-100–44)	20	-2 (-76–90)	27	0.712
**Respiratory rate (breaths per min)**	8 (-12–104)	33	4 (-4–104)	20	0 (-88–140)	27	0.088
**Temperature (°C)**	-0.1 (-2.2–2.5)	33	0.0 (-1.8–1.4)	20	-0.6 (-1.7–1.5)	26	0.130
**Triglycerides (mmol/l)**	4.9 (-0.2–117.0)	34	5.4 (-0.0–91.0)	18	9.4 (1.1–75.4)	28	0.041*
**pH**	0.01 (-0.26–0.19)	31	0.00 (-0.15–0.07)	17	0.01 (-0.10–0.30)	27	0.584
**pCO**_**2**_ **(mmHg)**	-4.4 (-33.6–14.4)	30	-4 (-16.8–21.2)	17	-8.4 (-32.6–4.3)	27	0.235
**HCO**_**3**_^**-**^ **(mmol/l)**	-3.1 (-10.9–4.9)	31	-1.2 (-9.2–4.4)	17	-3.9 (-1.6–3.0)	25	0.053
**BE**	-3 (-9–4)	31	0 (-10–2)	17	-4 (-10–6)	26	0.079
**Lactate (mmol/l)**	-0.1 (-5.8–2.6)	32	-0.3 (-3.5–8.7)	18	0.7 (-3.4–5.0)	25	0.034*
**Na (mmol/l)**	-3 (-12–6)	31	-2 (-6-3)	17	-4 (-11-5)	26	0.148
**K (mmol/l)**	-0.4 (-1.7–0.2)	31	0.0 (-1.4–0.7)	17	-0.4 (-2.3–2.7)	27	0.084
**iCa (mmol/l)**	-0.10 (-0.22–0,03)	31	-0.01 (-0.13–0.11)	17	-0.07 (-0.23–0.06)	27	0.002*
**Hb (mmol/l)**	0.0 (-5.8–8.5)	31	-0.3 (-4.4–11.5)	16	0.9 (-11.8–6.9)	2	0.893
**HCT (%)**	-2.0 (-23–25)	31	-1 (-13–34)	16	-1 (-35–23)	25	0.710

n: number of animals

*: significant changes; p: significance level; HCO_3_^-^: bicarbonate; BE: base excess; Na: sodium; K: potassium; iCa: ionised calcium; Hb: Hemoglobin; HCT: hematocrit; pCO_2_: carbon dioxide partial pressure.

In addition, the changes in the MGCS, physical examination parameters, and laboratory parameters three hours after discontinuing ILE were analyzed. Patients with worsening MGCS consistently showed a decrease in base excess (p <0.001) and a decrease in potassium (p = 0.022; Table 7).

**Table 7:** Course of physical examinations and laboratory parameters (triglycerides, lactate, venous blood gas analysis) between the administration and 3 hours after the end of intravenous lipid emulsion (ILE) for the treatment of conformed or suspected poisonings in 65 dogs and 17 cats in relation to the course of the modified Glasgow coma scale (MGCS) given as absolute number of pets.

Parameter	MGCS	MGCS	MGCS	p
	improved (34)	equal (20)	worsened (28)	
**Heart rate increase**	15/34	8/20	13/27	0.624
**Heart rate decrease**	17/34	10/20	14/27	
**Heart rate equal**	2/34	2/20	0/27	
**Respiratory rate increase**	25/33	12/20	13/27	0.058
**Respiratory rate decrease**	2/33	1/20	7/27	
**Respiratory rate equal**	6/33	7/20	7/27	
**Temperature increase**	14/33	8/20	5/26	0.161
**Temperature decrease**	18/33	9/20	19/26	
**Temperature equal**	1/33	3/20	2/26	
**Triglyceride increase**	32/34	16/18	28/28	0.229
**Triglyceride decrease**	2/34	2/18	0/28	
**Lactate increase**	15/32	7/18	17/25	0.126
**Lactate deacrease**	17/32	11/18	8/25	
**pH increase**	15/31	9/17	16/27	0.594
**pH decrease**	16/31	8/17	10/27	
**pH equal**	0/31	0/17	1/27	
**pCO**_**2**_ **increase**	6/30	5/17	5/27	0.667
**pCO**_**2**_ **decrease**	24/30	12/17	22/27	
**HCO**_**3**_^**-**^ **increase**	4/31	5/17	1/25	0.062
**HCO**_**3**_^**-**^ **decrease**	27/31	12/17	24/25	
**BE increase**	6/31	2/17	0/26	<0.001*
**BE decrease**	24/31	8/17	26/26	
**BE equal**	1/31	7/17	0/26	
**Na increase**	3/31	5/17	4/26	0.166
**Na decrease**	26/31	10/17	22/26	
**Na equal**	2/31	2/17	0/26	
**K increase**	6/31	6/17	9/27	0.022*
**K decrease**	23/31	6/17	17/27	
**K equal**	2/31	5/17	1/27	
**iCa increase**	4/31	7/17	3/27	0.100
**iCa decrease**	25/31	9/17	21/27	
**iCa equal**	2/31	1/17	3/27	
**Hb increase**	15/31	7/16	13/25	0.904
**Hb decrease**	15/31	9/16	11/25	
**Hb equal**	1/31	0/16	1/25	
**HCT increase**	13/31	9/16	14/25	0.651
**HCT decrease**	17/31	7/16	11/25	
**HCT equal**	1/31	0/16	0/25	

*: significant differences; p: significance level; HCO_3_^-^: bicarbonate; BE: base excess; Na: sodium; K: potassium; iCa: ionised calcium; Hb: Hemoglobin; HCT: hematocrit; pCO_2_: carbon dioxide partial pressure.

### Adverse effects

A total of 86 adverse effects were documented in 64 animals (78.0%) in median two hours (0.5–4.0 hours) after starting ILE. Almost 59 of all patients (71.9%) showed a deterioration in the level of consciousness during or within three hours after discontinuing ILE. The level of consciousness changed to apathetic in 15 patients (18.2%), stuporous in 23 patients (28.0%) and comatose in 21 patients (25.3%). In eight patients vomiting (9.7%), in 15 patients bradycardia (11.0%) and in three patients hypersalivation (3.6%) was observed less frequently (Table 8). Some described adverse effects have been attributed to prior administration of anticonvulsants due to a seizure event or administration of anesthetics in conjunction with prior gastric lavage. If patients already showed adverse effects before ILE administration not associated with ILE they were excluded from the count as well as those who showed decreased consciousness or bradycardia at the time of presentation.

**Table 8:** Frequency of occurrence of adverse effects in 65 dogs and 17 cats (82 animals) after treatment of poisoning with intravenous lipid emulsion (ILE).

Adverse effect	n	%
**Animals with adverse effects**	64	78.0
**Animals without adverse effects**	18	22.0
**Decreased level of consciousness**	59	71.9
**Apathy**	15	18.2
**Stupor**	23	28.0
**Coma**	21	25.3
**Vomiting**	8	9.7
**Hypersalivation**	3	3.6
**Premature ventricular contraction**	1	1.2
**Bradycardia**	15	11.0

n: number of animals; %: percentage of animals.

Adverse effects were likely to be associated with ILE in 38 dogs (46.3%) and 6 cats (7.3%). Half of the patients (50.0%) showed a deterioration in consciousness that could be attributed to the administration of ILE. A quarter (25.6%) of all patients became stuporous and 9.8% comatose. Vomiting was observed in one in ten patients (9.8%; Table 9). The latter only includes patients vomiting despite antiemesis during or after ILE administration or vomiting for the first time during or after ILE.

**Table 9:** Incidence of adverse effects likely associated with intravenous lipid emulsion (ILE) for treatment of poisoning in 65 dogs and 17 cats.

Adverse effect	Likely association with ILE	
	n	%
**Animals with adverse effects**	44	53.6
**Decreased level of consciousness**	41	50.0
**Apathy**	12	14.6
**Stupor**	21	25.6
**Coma**	8	9.8
**Vomiting **	8	9.8
**Hypersalivation**	3	3.7
**PCV**	1	1.2
**Bradycardia**	7	8.0

Bradycardia was defined as a heart rate <60 bpm in dogs and <120 bpm in cats. PCV: Premature ventricular contraction; n: number of animals; %: percentage of animals.

**Fig 1:**
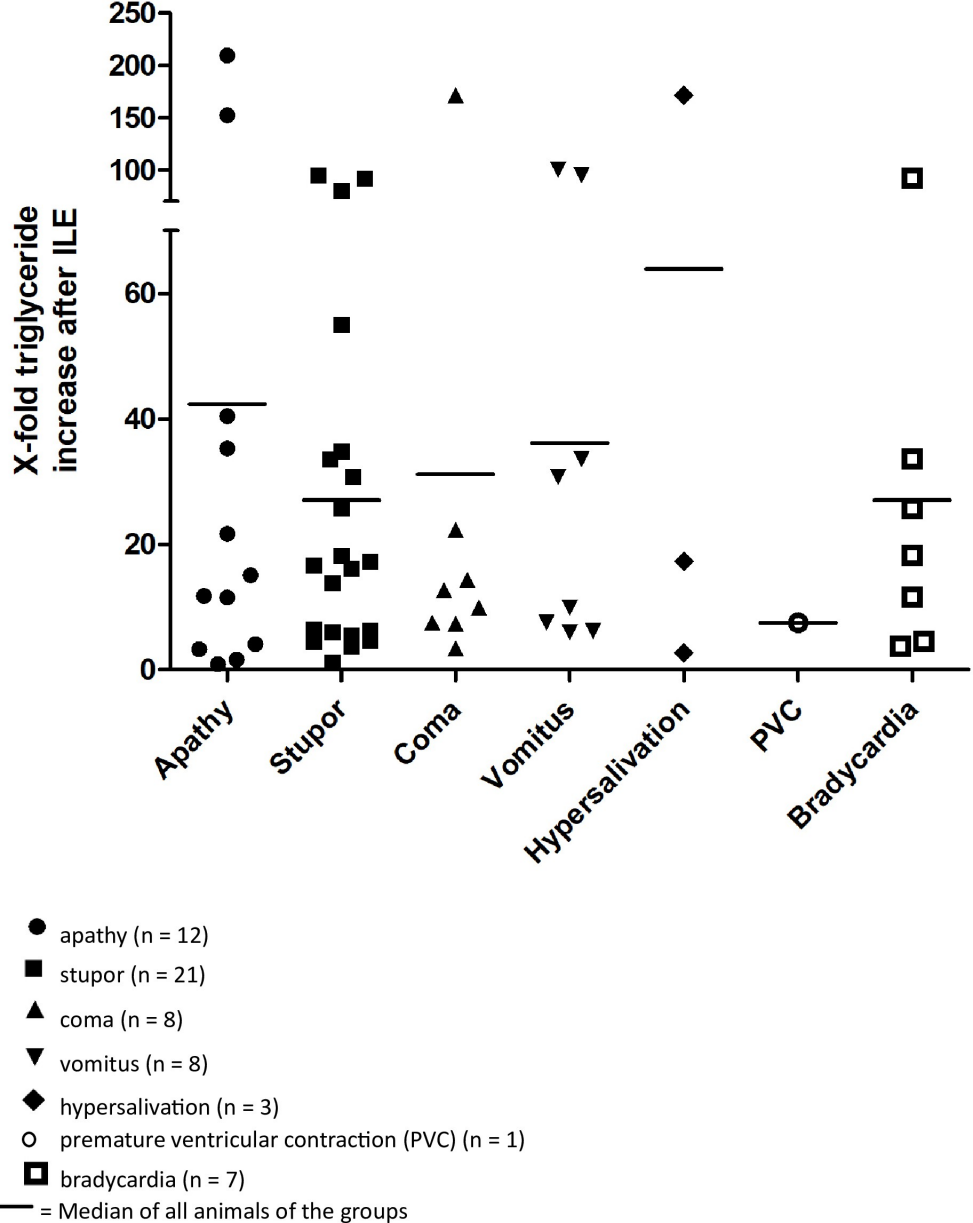
Occurrence of adverse effects in 82 poisoned animals (65 dogs and 17 cats) associated with a multiple increase in concentrations of serum triglyceride.

Worsening of the level of consciousness was associated with higher serum triglyceride levels three hours after ILE compared to the baseline value. Patients with neurologic adverse effects had an increase in triglyceride concentration (p < 0.001). In particular, comatose patients had increased triglyceride levels more than six times over reference interval. Vomitus was also associated with increased triglyceride levels compared to the baseline value (p = 0.008). In summary, patients with adverse effects had a total triglyceride increase of 8.1 mmol/l (-0.2–81.5 mmol/l) and those without adverse effects had a triglyceride increase of 5.2 mmol/l (-0.1–117.0 mmol/l; p = 0.191). No difference in triglyceride increase between the groups of adverse effects was found (Fig 1).

**Fig 2:**
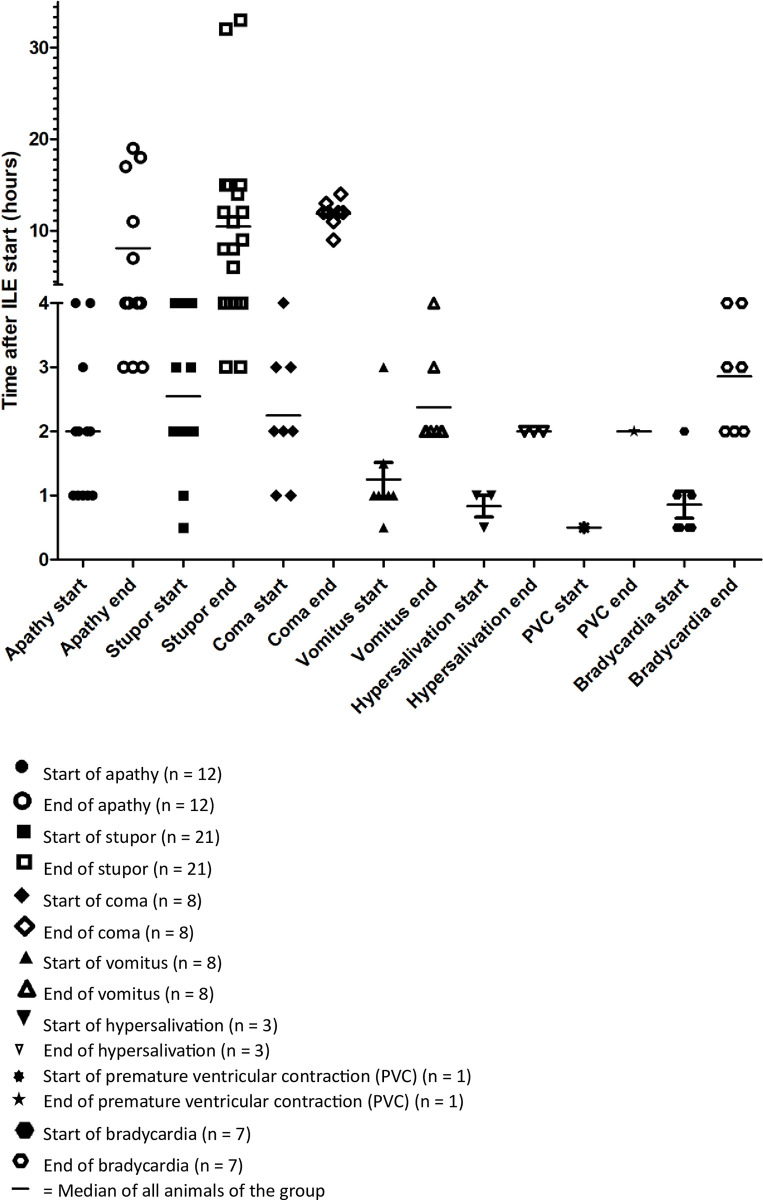
Time of occurrence of adverse effects during and within four hours after intravenous lipid emulsion (ILE) for the treatment of confirmed or suspected poisonings in 65 dogs and 17 cats and their reversibility.

Adverse effects occurred within four hours after the start of the ILE administration. Decreased level of consciousness occurred mainly within 1–3 hours after the start of ILE administration. Vomiting, hypersalivation, premature ventricular contractions and bradycardia were observed mainly within the first hour after initiation of ILE (Fig 2). Antiemetics were administered to 65.8% of patients before ILE administration due to initial vomitus, gastric lavage or induced emesis.

The complete reversal of all adverse effects occurred within 33 hours. No animal died due to the adverse effects. Reversal of the decreased level of consciousness occurred within 15 hours in 39 (95%) of the 41 affected animals. There was no difference in the duration of neurological adverse effects depending on the severity of the neurological adverse effects. Vomiting was no longer observed than four hours after the start of the ILE administration and hypersalivation after two hours (Fig 2).

## Discussion

In this retrospective analysis, 82 patients (65 dogs and 17 cats) were treated with the in-house protocol with ILE due to confirmed or suspected poisoning with a lipophilic substance. Multiple case reports exist in human medicine, but only a few in veterinary medicine. Therefore, to the authors’ knowledge, this study is the first one with both, a representative size of patients treated for poisonings with ILE according to a standard protocol and with a meaningful comparison of important laboratory findings and time-defined occurrence of effects and adverse effects of ILE therapy. All patients received the same dosage of ILE and each adverse effect was assessed for its temporal occurrence and in relation to the development of the laboratory results [21, 22, 27–30].

Both, dogs and cats, are mainly young animals, whereby dogs with 62 patients are represented significantly more often (79%) than cats in this study. The toxicant ingested was unknown in 51 (62.2%) of affected animals. These were treated with ILE due to severe clinical signs of poisoning. Regardless of the suspected toxicant, all patients received the same dosage of ILE over the same period, so the occurrence of potential adverse effects cannot be attributed to different total administered doses of ILE. A log P significantly >1.0 as in the cases of THC, carprofen, permethrin spot on or ivermectin are substances for which the use of ILE is indicated due to lipophilicity [1, 3, 14–16]. Some animals were presented with typical signs of poisoning such as hyperesthesia, twitching, ataxia and convulsions after ingestion of alpha-chloralose. The log P of alpha-chloralose is reported between 0.85 and 1.0 depending on the literature, so the administration and therefore the benefit of ILE in this type of poisoning is questionable [32, 33]. After data collection, a study by Tegner et al. showed that, unfortunately, ILE is actually of no benefit, so that in the administration should rather be refrained in the case of alpha-chloralose intake [34]. However, the decision to administer ILE was made by the clinician in charge in the emergency situation due to suspected poisoning often not being aware of the final toxicant. Ingestion of tremorgenic mycotoxin is toxic to animals, but no log P is known. However, patients also showed typical signs of poisoning such as tremors, ataxia and hyperesthesia, and there are several case reports of successful treatment with ILE [35, 36]. There is a wide range of different and often unknown toxicants. A toxicological screening was usually not desired by the owners for monetary reasons so there is no absolute certainty of the toxicant. This makes a certain variability of the effect of ILE likely.

Half of the patients (50%) showed a clouding of consciousness that could be reliably linked to the administration of ILE, which is a high number of patients affected. Most frequently, a stuporous state in 21 patients (25.6%) followed by apathy in 12 patients (14.6%) and a comatose state in eight patients (9.8%) was observed. Vomiting and bradycardia were equally common [37, 38]. Rarely hypersalivation and premature ventricular contractions were observed. Less than 5.8% of patients treated with ILE due to poisonings had adverse effects [30]. This study suggests adverse effects of ILE occur more frequently than previously assumed.

The temporal occurrence of the adverse effects described shows that vomiting, hypersalivation, premature ventricular contraction and bradycardia occur mainly within the first hour and thus during or shortly after cessation of ILE administration. In these patients, triglycerides are often increased more than six-fold in the control after three hours, and in some even more than thirty-fold. This shows that there may be a correlation between patients with a more than six-fold increase in blood triglycerides and the occurrence of acute and temporary adverse effects. In human medicine, the phenomenon of rapid responses of vomiting and tachypnoea has also been associated with the rapid administration of a large amount of ILE. A fat overload syndrome was suspected, which is also conceivable in animals [21, 39]. A correlation between race, age or sex and adverse effects could not be observed in this study. A rapid decline in adverse effects after discontinuation of therapy described in previous case reports was also observed [21]. Within two hours, the clinical signs of vomiting, hypersalivation, premature ventricular contraction and bradycardia adverse effects were reversible in most animals. This could potentially be associated with an acute reaction of the body to the rapid administration of a large amount of ILE.

A decreased level of consciousness, on the other hand, was observed mainly within one to three hours after the start of ILE administration and thus later than vomiting, for example. Simultaneously with a decrease in the state of consciousness—up to coma—a decrease in the signs of poisoning such as hyperesthesia, tremor, convulsions and ataxia was also observed, and even after complete recovery of normal consciousness, these clinical signs were usually no longer observed. The decrease in symptoms of poisoning supports the "lipid shuttle" theory to remove lipophilic toxicants, but this theory does not explain the decrease in consciousness [14, 36]. It was observed that there was a significant difference between patients with worsening MGCS and an increase in blood triglycerides compared to those with improvement in MGCS. In the former, there was a significant increase in triglycerides. This was often six-fold to thirtyfold increased, although in isolated cases, clouding of consciousness also occurred with a slight increase.

In summary, there seems to be a correlation for patients with adverse effects having a higher triglyceride increase than patients without. Daza Gonza’les et al. observed increased triglycerides after the administration of ILE, and individual case reports also suggest a correlation with diminished consciousness [22, 28, 31].

Due to the rapid increase of triglycerides in the blood, a fat overload syndrome is conceivable. At the same time an increase in lactate in the blood, which suggests a perfusion problem and thus tissue hypoxia, was also observed. In addition, the bicarbonate drops in the affected patients, which, in connection with a decrease in the base excess, suggests a lack of bases in the blood and metabolic acidosis. It should be noted, however, that the difference in bicarbonate is not significant. A deterioration of the state of consciousness, on the other hand, is mainly observed within one to three hours after the start of ILE administration and the latter was also observed in two case reports in human medicine and attributed to a fat overload syndrome [21, 40]. Theoretically, it is conceivable that the rapid administration of a large amount of ILE within a short time and the accompanying increase in triglycerides in the blood lead to increased viscosity. This can lead to decreased tissue perfusion and can be associated with tissue hypoxia. This probably occurs not only in the periphery but also in the central organs, leading to a temporary clouding of consciousness. There is also the complication of a fat embolus in the lungs, which was described in a case report of a child [41]. Although a study on rabbits shows ILE to be histologically and biochemically safe, a fat embolism in the brain cannot be excluded and is therefore also conceivable [17].

A decrease in clouding of consciousness is observed in the third hour after administration. More than half of the affected apathetic patients are clinically unremarkable after four hours and those with stupor and coma after eight to twelve hours. Complete resolution of clinical signs in all patients is observed after no more than twenty hours in those with apathy and coma and, after no more than 33 hours, in those with stupor. The time course suggests that there must be a significant accumulation of triglycerides in the blood before clouding of consciousness occurs. This means doubling at least. In addition, a significantly longer time is required before almost complete metabolism has taken place and the adverse effects were reversible.

In human medicine, these adverse effects have often been associated with tachypnoea, tachycardia or hyperthermia [22, 25, 39]. In this study, only an increase in temperature of 0.4°C on average over twelve hours was recorded. No significant difference in physical examination findings was observed between patients with improvement or worsening of MGCS.

A decrease in the levels of electrolytes potassium, sodium and calcium was observed in both improvement and deterioration of the MGCS. No variation in the electrolytes was observed if the MGCS was stable. This leads to the suggestion that there is a relationship between the decrease of electrolytes and changes in consciousness. However, the values were almost all still within the reference range. At this point, a dilution effect through the administration of a large amount of fluid due to ILE therapy is favored. Hypocalcemia was present in the only animal that died. Holowaychuk et al. found that hypocalcemia was more common in critically ill patients suggesting initial critical circumstances with a worse prognosis [42]. However, this would need to be re-evaluated by a larger study.

Other adverse effects such as reddening of the skin, pruritus, corneal lipidosis, acute respiratory distress syndrome or even swelling and pain at the entry site of the peripheral venous catheter were not observed in this study [5, 8, 16, 18, 20].

If signs of poisoning reappear or persist after the first administration of ILE, the concentration of triglycerides in the blood should always be measured since significantly increased triglycerides could potentially lead to a higher risk of adverse effects, which need to be proved in further studies.

Irrespective of the suspected poisoning and the severity of the clinical signs, the administration of ILE led to a rapid improvement in the neurological status, which was documented in the form of the MGCS. With early intervention in the case of poisoning, symptoms usually regress with adequate treatment. However, this can probably be accelerated by the ILE administration [30]. This is also reflected in the study by Peacock et al., in which morbidity was reduced by the administration of ILE in cats with permethrin poisoning [16]. Hospitalization time in the current study averaged 17 hours in dogs and 25 hours in cats. The reason for the shorter time in dogs could be the earlier time of presentation after ingestion of toxicant and thus faster treatment with decontamination. In addition, the outcome of ILE treatment after poisoning is good regardless of the severity of clinical signs at presentation and the occurrence and extent of adverse effects [30]. Almost all but one patient survived and did not show clinical signs of poisoning at the time of discharge.

The American College of Medical Toxicology (ACMT) does not give a standard requirement for the administration of ILE nor reasons against its use. However, in cases of serious hemodynamic instability due to xenobiotics with high lipophilicity, it is considered to be a useful therapeutic option. ILE can also be useful for other medication poisonings in which standard resuscitation measures fail. Thus, in this statement, its use is even recommended only in cases of hemodynamic instability.

In this study, ILE administration did not show any lasting negative effects. The described adverse effects were all reversible in a short period, which was also shown in other studies [30]. However, considering the statement of the ACMT and the frequency of adverse effects, ILE administration should be thoroughly considered by the treating veterinarian [30, 43, 44].

## Limitations

The limitation of this retrospective study is on the one hand the large number of unknown poisonings due to which a certain variability of the effect is to be expected despite the same administration duration and quantity of ILE. A more detailed and more tangible statement on the effect of ILE in individual poisonings would have been possible employing a toxicological screening. In this case, it might also have been possible to make a statement about the connection with possible adverse effects. On the other hand, the data sets are partly incomplete. A prospective study with complete data sets and close monitoring every 15 minutes during ILE administration including cardiovascular and other hemodynamic parameters concerning blood pressure and ECG would have been desirable as recommended by the ACMT.

## Conclusion

Overall, neurological status improved, hospitalization time was relatively short, and the outcome was good. However, half of the patients in this retrospective study experienced adverse effects of varying severity, so the benefits should be carefully considered before administering ILE. Increased triglycerides may be associated with an altered level of consciousness. Long-term studies with assessment of blood pressure and ECG to assess cardiovascular status and perfusion during and after administration of ILE, as well as performing a toxicological screening to accurately define poisoning, are advisable.
